# Alum Catalyzed Simple, Efficient, and Green Synthesis of 2-[3-Amino-5-methyl-5-(pyridin-3-yl)-1,5-dihydro-4*H*-1,2,4-triazol-4-yl]propanoic Acid Derivatives in Aqueous Media

**DOI:** 10.1155/2013/716389

**Published:** 2013-10-29

**Authors:** Harshita Sachdeva, Diksha Dwivedi, Rekha Saroj

**Affiliations:** Department of Chemistry, Faculty of Engineering and Technology, Mody Institute of Technology and Science, Lakshmangarh, Sikar, Rajasthan 332311, India

## Abstract

Alum (KAl(SO_4_)_2_ *·*12H_2_O) is an inexpensive, efficient, and nontoxic catalyst used for the synthesis of 2-[3-amino-5-methyl-5-(pyridin-3-yl)-1,5-dihydro-4*H*-1,2,4-triazol-4-yl]propanoic acid derivatives in aqueous media by the reaction of 3-acetyl pyridine **(1)**, amino acids **(2)**/**(6)**, and thiosemicarbazide **(4)** at 80°C. This methodology offers significant improvements for the synthesis of products with regards to the yield of products, simplicity in operation, and green aspects by avoiding toxic catalysts which uphold the motto of green chemistry. Synthesized compounds have been characterized by FT-IR, ^13^C NMR, and ^1^HNMR spectroscopy.

## 1. Introduction

In the last two decades, it has become increasingly clear that the chemical industries are facing serious environmental problems. Many of the classical synthetic methodologies have a broad scope but generate copious amounts of waste, and the chemical industry has been subjected to increasing pressure to minimize or, preferably, eliminate this waste. Many organic solvents are hazardous and can be deleterious to human health. They are volatile and cause an environmental threat by polluting the atmosphere. Green chemistry approaches are significant due to reduction in byproducts, waste produced, and energy cost. In recent years, organic research is mainly focused on the development of green methods which involve the use of alternative reaction media to replace volatile and hazardous solvents commonly used in organic synthesis. In this regard, the use of water as a reaction solvent has also attracted great attention and has become an active area of research in green chemistry.

Nowadays, many organic transformations have been carried out in water [1–3]. It is a unique solvent due to being readily available, inexpensive, nontoxic, safer, and environmentally benign. The aqueous mediated conditions lead to enhanced reaction rates, higher yields of pure products, and easier workup and sometimes to selective conversions with several advantages of the ecofriendly approach in the framework of green chemistry. Consequently, this protocol should be welcomed in these environmentally conscious days.

Catalysis cannot only help to green chemical processes (e.g., by replacing reagents or by enabling more efficient processes), but the demonstration of their value to reduce the environmental impact of processes and reduce the costs of the processes will catalyze the greening of chemistry. In recent years, alum (KAl(SO_4_)_2_·12H_2_O) is extensively used as a catalystin organic synthesis because it is a nontoxic, inexpensive, ecofriendly, and easy handling catalyst. Other advantages include mild acidity, involatility, incorrositivity, insolubility in common organic solvents, and so forth. Alum has previously been reported to be effective in the synthesis of 1,4-dihydropyridines [4], cis-isoquinolic acids [5], mono- and disubstituted 2,3-dihydroquinazolin-4(1H)-ones [6], dihydropyrimidine via Biginelli reaction [7], coumarins [8], 5-arylidene-2,4-thiazolidinedione [9], dibenzoxanthenes [10], 1,5-benzodiazepines [11], and trisubstituted imidazoles [12]. We investigated alum as a catalyst for the synthesis of 2-[3-amino-5-methyl-5-(pyridin-3-yl)-1,5-dihydro-4*H*-1,2,4-triazol-4-yl]propanoic acid derivatives **(5a–i)** by the reaction of 3-acetyl pyridine and amino-acids with thiosemicarbazide.

The search for new agent is one of the most challenging tasks to the medicinal chemist. The synthesis of high nitrogen containing heterocyclic system has been attracting increasing interest because of its utility in various applications, such as propellants, explosives, pyrotechnics, and especially chemotherapy. There is real perceived need for the discovery of new compounds endowed with antimicrobial activity, possibly acting through mechanisms of action, which are distinct from those of well-known classes of antibacterial agents to which many clinically relevant pathogens are now resistant. 1,2,4-Triazoles represent an overwhelming and rapid developing field in modern heterocyclic chemistry. 

**Scheme 1:**
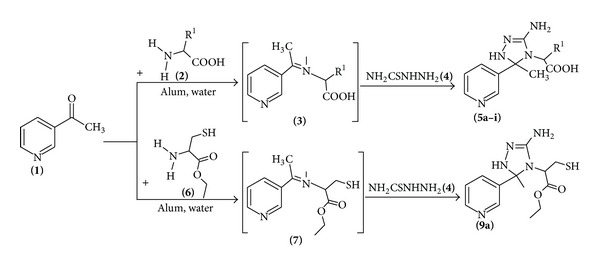


From the literature, it is predictable that 1,2,4-triazoles represent important pharmacophores and play a vital role as medicinal agents. A degree of respectability has been bestowed for 1,2,4-triazole derivatives due to their wide range of biological activities such as antimicrobial [13–15], analgesic, anti-inflammatory [16, 17], anticancer [18] and antioxidant properties [19]. Ribavirin (antiviral) [20] rizatriptan (antimigraine) [21], posaconazole, fluconazole, and Itraconazole [22, 23] are efficient antifungal drugs currently used in the treatment of fungal infection. Several articles devoted to the synthesis and biological activity of arylidenaminotriazoles have been published [24–29] due to their potential biological activities. In view of the above observations, an attempt has been made to synthesise 2-[3-amino-5-methyl-5-(pyridin-3-yl)-1,5-dihydro-4*H*-1,2,4-triazol-4-yl]propanoic acid derivatives **(5a–i)/(9a)** (Scheme 1) by treating 3-acetyl pyridine **(1)** and amino acids **(2)/(6)** with thiosemicarbazide **(4)** under the framework of green chemistry using alum as a green catalyst in water.

## 2. Results and Discussion

In continuation to our work on the development of green methodologies [30–32] for heterocyclic synthesis, herein we would like to report a simple, efficient, and rapid method for the synthesis of triazole derivatives **(5a–i)/(9a)** (Table 1). The condensation of 3-acetyl pyridine with various amino acids/cysteine amino acid yielded 2-{[1-(pyridin-3-yl)ethylidene]amino}propanoic acid **(3)**/ethyl 2-{[1-(pyridin-3-yl)ethylidene]amino}-3-sulfanylpropanoate **(7)** which “insitu” were cyclocondensed with thiosemicarbazide **(4)** in the presence of alum in aqueous medium to give exclusively **5a–i/9a**, respectively, in 86–90% (Scheme 1) (Table 1). We have recently reported [33] the environ-economic synthesis of some new 1,2,4-triazole derivatives by the reaction of 4-chloro-2-nitro aniline and aromatic aldehydes with thiosemicarbazide in high yields and shorter reaction time in the presence of lemon juice as a green catalyst.

 The reaction was extensively studied under different reaction conditions to find out the best method giving the product in higher yield and short reaction time with operational simplicity. As shown in Tables 2, 3, and 4, the reaction of 3-acetyl pyridine **(1)** and alanine amino acid **(2)** with thiosemicarbazide **(4)** was chosen as the model substrate to optimize reaction conditions including effect of temperature, type of catalyst, and concentration of catalyst.  Table 2 shows the effect of temperature on the formation of compound **5a** in the presence of alum as a green catalyst. It is observed that the rate of reaction increases on increasing the temperature due to increase in acidity and the best yield is obtained at 80°C temperature in a short reaction time of 6 hrs. The acidity of the alum depends highly on the quantity of trapped water molecules in the interlayers. Alums liquefy on heating and if the heating is continued, the water of crystallization is driven off, the salt froths and swells, causing decrease in Bronsted acidity but increase in Lewis acidity. Hence other compounds were also synthesized under similar reaction conditions (at 80°C).

 In Table 3, our results are compared with results obtained by other catalysts for the synthesis of compound **5a**. The data presented in this Table 3 shows the promising features of this method in terms of the yield of the product compared to other catalysts. Other catalysts, namely, silica, alumina, and phosphorus pentoxide were also screened at 80°C (Table 3) (entries **1–3**), and the results show that the alum provided the highest yield (entry **4**) (Table 3). Notably, a very slow reaction was observed when the catalytic amount of alum was decreased from 15 to 10 mol% (entry **2** versus entry **1**) (Table 4). When the catalytic amount of alum is increased from 15 to 20 mol%, a large increase in yield is observed (entry **2** versus entry **3**). With 25 to 30 mol% of alum, there is no change in reaction rate as well as yield of the product (entry **4** versus entry **5**). Further, there is an increase in 4% yield when mol% of alum is increased from 20 to 25% (entry **3** versus entry **4**).

 Herein, we have developed an efficient methodology for the synthesis of triazole derivatives** (5a–i)** using alum as a green catalyst in aqueous medium at 80°C. The methodology developed is simple giving product in excellent yields. To investigate the generality of the reaction, various substituted amino acids were studied, all of which undergo smooth reactions without the formation of any byproduct (Table 1) as observed on TLC.

## 3. Experimental

Chemicals were purchased from Sigma-Aldrich and Merck and used without further purification. Melting points were determined on an Instrument India Melting Point Apparatus. The spectral analyses of synthesized compounds have been carried out at SAIF, Punjab University, Chandigarh. Monitoring the reactions and checking the purity of the final products were carried out by thin layer chromatography (TLC) on silica gel G plates using benzene : ethyl acetate (7 : 3 v/v) as eluent. IR spectra were recorded in KBr on a Perkin Elmer Infrared L1600300 Spectrum Two Li Ta spectrophotometer. ^1^H and ^13^C NMR spectra were recorded on Bruker Avance II 400 NMR Spectrometer using DMSO as solvent and tetramethylsilane (TMS) as internal reference standard. The obtained products were identified from their spectral (^1^H NMR, ^13^C NMR and IR) analyses.

### 3.1. General Procedure for the Synthesis of 2-[3-Amino-5-methyl-5-(pyridin-3-yl)-1,5-dihydro-4*H*-1,2,4-triazol-4-yl]propanoic Acid Derivatives (**5a–i**)/**9a**

The compounds were synthesized by the reaction of 3-acetyl pyridine and various amino acids with thiosemicarbazide in aqueous medium using alum as an ecofriendly catalyst. In a round bottom flask was placed a mixture of 3-acetyl pyridine **1** (2 mmol), amino acid **2** (2 mmol), and alum (25 mol%) in water (25 mL). The suspension was stirred at 80°C for a certain period of time required to complete the reaction (as monitored by TLC). As the reactants disappeared, 2 mmol of thiosemicarbazide was added and again stirred at 80°C for appropriate time. After the completion of reaction, the obtained product was filtered, washed with cold water, and recrystallized from ethanol.

### 3.2. Characterization of the Compounds is Carried out on the Basis of Spectral Data

#### 3.2.1. **5a**. 2-[3-Amino-5-methyl-5-(pyridin-3-yl)-1,5-dihydro-4*H*-1,2,4-triazol-4-yl]propanoic Acid

IR (KBr, cm^−1^) 3348, 3258, 2854, 2560, 1670, 1546, 1416, 1343, 1187, 1064, 747. ^1^H NMR (400 MHz, DMSO-d_6_): *δ* 1.25 (s, 3H, CH_3_), 1.53 (d, 3H, CH_3_), 3.88 (q, 1H, CH), 4.33 (s, 2H, NH_2_), 7.00 (s, 1H, NH), 7.58–8.60 (m, 4H, Ar–H), 11.10 (s, 1H, OH) ppm; ^13^C NMR (100 MHz, DMSO-d_6_): *δ* 26.16, 27.30, 42.68, 72.35, 99.49, 121.21, 123.74, 136.02, 146.08, 158.55, 174.67 ppm. Anal. Calcd. for C_11_H_15_N_5_O_2_: C, 53.00, H, 6.07, N, 28.10. Found: C, 52.82, H, 6.09, N, 28.07.

#### 3.2.2. **5b**. 2-[3-Amino-5-methyl-5-(pyridin-3-yl)-1,5-dihydro-4*H*-1,2,4-triazol-4-yl]butanedioic Acid

IR (KBr, cm^−1^) 3386, 3263, 3034, 2634, 1610, 1504, 1270, 1089, 926, 704. ^1^H NMR (400 MHz, DMSO-d_6_): *δ* 1.52 (s, 3H, CH_3_), 3.03 (d, 2H, CH_2_), 3.80 (t, 1H, CH), 4.21 (s, 2H, NH_2_), 7.15 (s, 1H, NH), 760–8.65 (m, 4H, Ar–H), 10.82 (s, 2H, OH) ppm; ^13^C NMR (100 MHz, DMSO-d_6_): *δ* 30.40, 37.41, 49.21, 68.10, 121.38, 125.40, 136.14, 145.42, 148.14, 157.45, 174.51, 177.04 ppm. Anal. Calcd. for C_12_H_15_N_5_O_4_: C, 49.14, H, 5.16, N, 23.88. Found: C, 48.94, H, 5.18, N, 23.86.

#### 3.2.3. **5c**. 2-[3-Amino-5-methyl-5-(pyridin-3-yl)-1,5-dihydro-4*H*-1,2,4-triazol-4-yl]-3-methyl Pentanoic Acid

IR (KBr, cm^−1^) 3371, 3264, 2967, 2619, 1608, 1586, 1394, 1187, 1089, 710.^ 1^H NMR (400 MHz, DMSO-d_6_): *δ* 1.04 (t, 3H, CH_3_), 1.10 (d, 3H, CH_3_), 1.68 (s, 3H, CH_3_), 2.58 (m, 1H, CH), 3.03 (m, 2H, CH_2_), 3.88 (d, 1H, CH), 4.55 (s, 2H, NH_2_), 7.00 (s, 1H, NH), 7.68–8.78 (m, 4H, Ar–H), 11.10 (s, 1H, OH) ppm; ^13^C NMR (100 MHz, DMSO-d_6_): *δ* = 13.42, 16.32, 24.01, 28.75, 32.45, 49.47, 69.65, 120.30, 124.00, 136.42, 145.78, 148.36, 157.74, 177.50 ppm. Anal. Calcd. for C_14_H_21_N_5_O_2_: C, 57.71, H, 7.27, N, 24.04. Found: C, 57.49, H, 7.26, N, 24.07.

#### 3.2.4. **5d**. 2-[3-Amino-5-methyl-5-(pyridin-3-yl)-1,5-dihydro-4*H*-1,2,4-triazol-4-yl]-3-(1H-imidazol-4-yl)propanoic Acid

IR (KBr, cm^−1^) 3386, 3127, 2880, 2710, 1634, 1480, 1342, 1251, 1086, 923, 704. ^1^H NMR (400 MHz, DMSO-d_6_): *δ* 1.83 (s, 3H, CH_3_), 3.42 (t, 1H, CH), 2.58 (d, 2H, CH_2_), 4.34 (s, 2H, NH_2_), 7.40–7.93 (s, 2H, NH), 7.34–9.03 (m, 6H, Ar–H), 10.19 (s, H, OH) ppm; ^13^C NMR (100 MHz, DMSO-d_6_): *δ* 29.58, 30.45, 52.10, 70.46, 110.24, 121.38, 122.45, 125.47, 136.14, 144.42, 147.21, 157.78, 177.45 ppm. Ms: *m/z*: 315. Anal. Calcd. for C_14_H_17_N_7_O_2_: C, 53.32, H, 5.43, N, 31.09. Found: C, 53.93, H, 5.45, N, 31.74.

#### 3.2.5. **5e**. 2-[3-Amino-5-methyl-5-(pyridin-3-yl)-1,5-dihydro-4*H*-1,2,4-triazol-4-yl]-4-(methyl sulfanyl)butanoic Acid

IR (KBr, cm^−1^) 3386, 3263, 2917, 2610, 1611, 1506, 1494, 1089, 975, 704. ^1^H NMR (400 MHz, DMSO-d_6_): *δ* 1.94 (s, 3H, CH_3_), 1.96 (s, 3H, CH_3_), 2.06 (m, 2H, CH_2_), 2.55 (d, 2H, CH_2_), 3.48 (t, 1H, CH), 4.65 (s, 2H, NH_2_), 7.10 (s, 1H, NH), 7.66–8.67 (m, 4H, Ar–H), 10.22 (s, 1H, OH) ppm; ^13^C NMR (100 MHz, DMSO-d_6_): *δ* 14.81, 26.06, 29.03, 30.45, 49.47, 66.78, 124.47, 136.56, 147.30, 145.45, 156.41, 177.54 ppm. Anal. Calcd. for C_13_H_19_N_5_O_2_S: C, 50.47, H, 6.19, N, 22.64. Found: C, 50.28, H, 6.18, N, 22.67.

#### 3.2.6. **5f**. 2-[3-Amino-5-methyl-5-(pyridin-3-yl)-1,5-dihydro-4*H*-1,2,4-triazol-4-yl]-3-phenyl Propanoic Acid

IR (KBr, cm^−1^) 3402, 3202, 2545, 1611, 1455, 1315, 1098, 856, 739. ^1^H NMR (400 MHz, DMSO-d_6_): *δ* 1.24 (s, 3H, CH_3_), 2.36 (d, 2H, CH_2_), 2.58 (t, 1H, CH), 4.92 (s, 2H, NH_2_), 7.23 (s, 1H, NH), 7.48–9.19 (m, 9H, Ar–H), 10.28 (s, 1H, OH) ppm; ^13^C NMR (100 MHz, DMSO-d_6_): *δ* 30.04, 35.37, 51.52, 65.12, 123.04, 125.45, 127.54, 128.65, 135.41, 139.08, 147.25, 140.36, 149.32, 154.23, 177.90 ppm. Anal. Calcd. for C_17_H_19_N_5_O_2_: C, 62.75, H, 5.89, N, 21.52. Found: C, 62.55, H, 5.91, N, 21.48.

#### 3.2.7. **5g**. 2-[3-Amino-5-methyl-5-(pyridin-3-yl)-1,5-dihydro-4*H*-1,2,4-triazol-4-yl]-3-hydroxy Propanoic Acid

IR (KBr, cm^−1^) 3421, 3320, 3264, 2879, 2603, 1647, 1556, 1354, 1207, 1075, 710. ^1^H NMR (400 MHz, DMSO-d_6_): *δ* 1.50 (s, 3H, CH_3_), 3.58 (t, 1H, CH), 3.90 (d, 2H, CH_2_), 4.71 (s, 2H, NH_2_), 7.08 (s, 1H, NH), 7.42–8.75 (m, 4H, Ar–H), 9.50 (s, 1H, OH), 11.05 (s, 1H, OH) ppm; ^13^C NMR (100 MHz, DMSO-d_6_): *δ* 30.21, 52.45, 65.02, 123.09, 135.14, 139.20, 147.34, 149.40, 154.03, 176.21 ppm. Anal. Calcd. for C_11_H_15_N_5_O_3_: C, 49.81, H, 5.70, N, 26.40. Found: C, 49.58, H, 5.68, N, 26.42.

#### 3.2.8. **5h**. 2-[3-Amino-5-methyl-5-(pyridin-3-yl)-1,5-dihydro-4*H*-1,2,4-triazol-4-yl]-3-(1H-indol-3-yl)propanoic Acid

IR (KBr, cm^−1^) 3402, 3202, 2545, 1611, 1481, 1315, 1056, 739. ^1^H NMR (400 MHz, DMSO-d_6_): *δ* 1.10 (s, 3H, CH_3_), 2.90 (d, 2H, CH_2_), 3.82 (t, 1H, CH), 4.82 (s, 2H, NH_2_), 7.10 (s, 1H, NH), 7.20–8.65 (m, 9H, Ar–H), 9.85 (s, 1H, NH), 10.48 (s, 1H, OH) ppm; ^13^C NMR (100 MHz, DMSO-d_6_): *δ* 28.41, 30.64, 56.21, 70.12, 110.04, 111.12, 118.14, 120.10, 121.16, 122.45, 124.40, 127.23, 136.20, 147.45, 154.24, 158.60, 174.57 ppm. Anal. Calcd. for C_19_H_20_N_6_O_2_: C, 62.62, H, 5.53, N, 23.06. Found: C, 62.40, H, 5.51, N, 23.09.

#### 3.2.9. **5i**. 2-[3-Amino-5-methyl-5-(pyridin-3-yl)-1,5-dihydro-4*H*-1,2,4-triazol-4-yl]-3-methyl Butanoic Acid

IR (KBr, cm^−1^) 3356, 3247, 2817, 2634, 1611, 1583, 1374, 1177, 1059, 983, 708. ^1^H NMR (400 MHz, DMSO-d_6_): *δ* 1.01 (d, 6H, 2 × CH_3_), 1.52 (s, 3H, CH_3_), 2.39 (m, 1H, CH), 3.48 (d, 1H, CH), 4.65 (s, 2H, NH_2_), 7.00 (s, 1H, NH), 7.42–7.88 (m, 4H, Ar–H), 11.02 (s, 1H, OH) ppm; ^13^C NMR (100 MHz, DMSO-d_6_): *δ* 16.91, 25.23, 30.24, 65.47, 123.10, 135.42, 139.10, 147.32, 154.06, 177.20 ppm. Anal. Calcd. for C_13_H_19_N_5_O_2_: C, 56.30, H, 6.91, N, 25.25. Found: C, 56.22, H, 6.92, N, 25.22.

#### 3.2.10. **9a**. Ethyl-2-[3-amino-5-methyl-5-(pyridin-3-yl)-1,5-dihydro-4*H*-1,2,4-triazol-4-yl]-3-sulfanylpropanoate

IR (KBr, cm^−1^) 3311, 3217, 2937, 1618, 1556, 1373, 1198, 1089, 735. ^1^H NMR (400 MHz, DMSO-d_6_): *δ* 1.30 (t, 3H, CH_3_), 1.24 (s, 1H, S–H), 1.52 (s, 3H, CH_3_), 3.03 (s, 2H, CH_2_), 3.77 (s, 1H, CH), 4.14 (q, 2H, CH_2_), 4.85 (s, 2H, NH_2_), 7.08 (s, 1H, NH), 7.42–7.88 (m, 4H, Ar–H) ppm; ^13^C NMR (100 MHz, DMSO-d_6_): *δ* 13.64, 25.32, 30.45, 51.45, 59.42, 64.23, 123.14, 135.40, 139.30, 147.45, 149.10, 154.20, 173.15 ppm. Anal. Calcd. for C_13_H_19_N_5_O_2_S: C, 50.47, H, 6.19, N, 22.64. Found: C, 50.27, H, 6.23, N, 22.67.

## 4. Conclusion

The use of water as a green solvent and alum as a green catalyst offers a convenient, nontoxic, inexpensive approach for the synthesis of triazole derivatives. This procedure is simpler, economical, milder, and faster, including cleaner reactions, high yields of products, and a simple experimental and workup procedure, which makes it a useful and attractive process and is also consistent with the green chemistry theme which affords good yields.

## Figures and Tables

**Table 1:** Physical characterization data of compounds **(5a–i)** and **(9a)**.

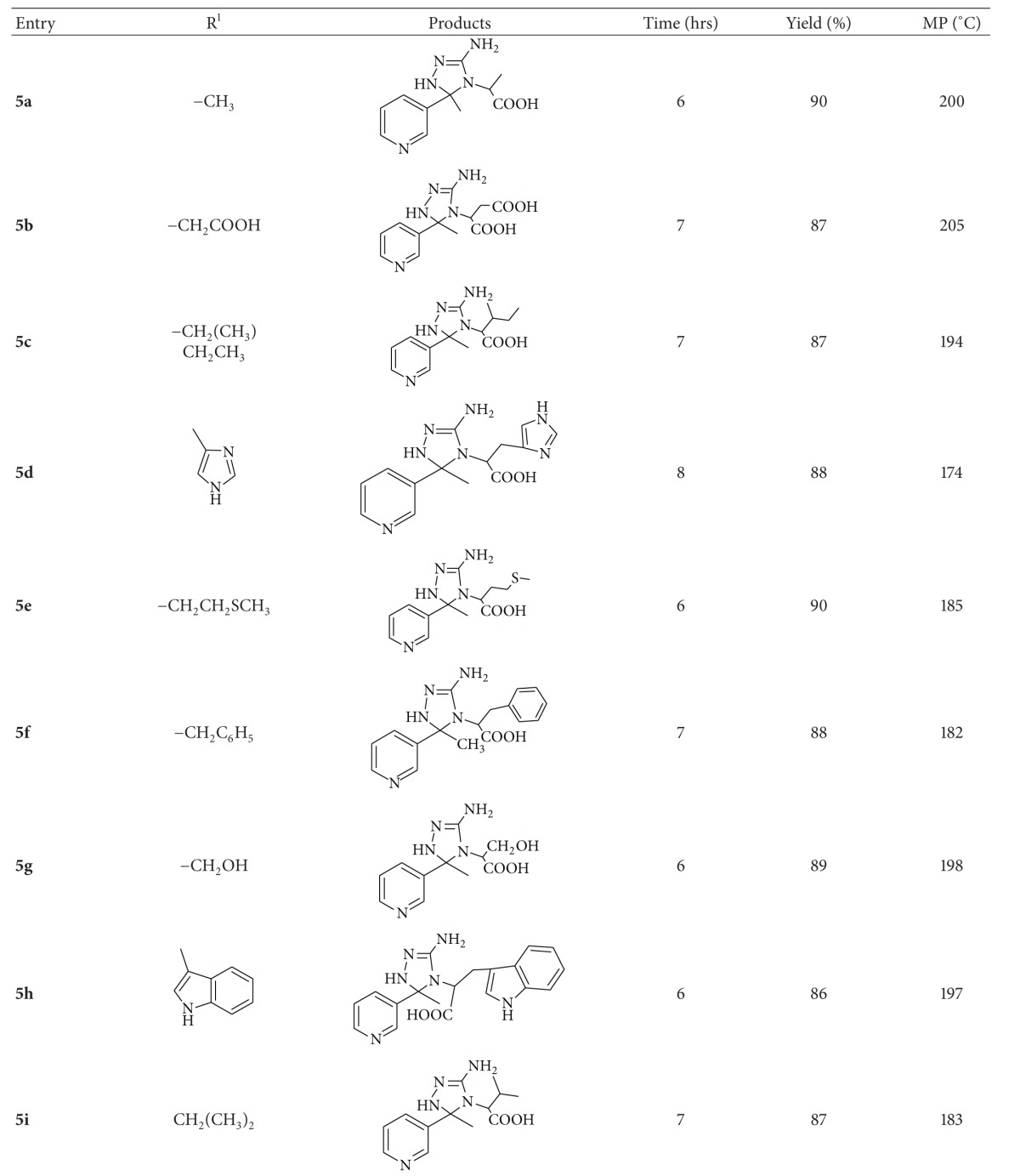 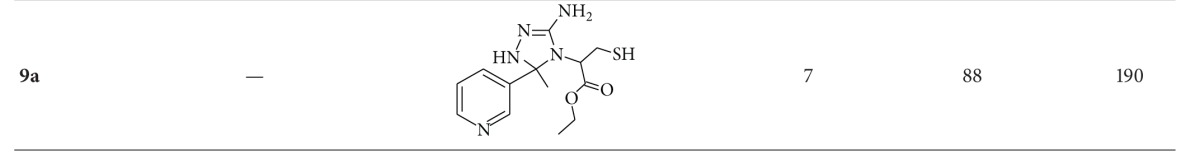

***Reaction Conditions:*** All the compounds were synthesized by stirring 3-acetyl pyridine **1** (2 mmol), amino acid **2** (2 mmol), thiosemicarbazide (2 mmol), and alum (25 mol%) in water (25 mL) at 80°C for 6-7 hrs.

**Table 2:** Effect of temperature on the formation of **5a** in the presence of alum as catalyst.

Entry	Temperature (°C)	Time (hrs)	Yield (%)
**1**	Room temp.	25	Nil
**2**	50	18	40
**3**	60	15	56
**4**	70	10	66
**5**	80	6	90
**6**	100	6	90

**Table 3:** Effect of various catalysts on the formation of **5a** at 80°C.

Entry	Catalyst	Time (hrs)	Yield (%)
**1**	SiO_2_	15	40
**2**	Al_2_O_3_	10	63
**3**	P_2_O_5_	8	75
**4**	Alum	6	90

**Table 4:** Effect of alum catalyst loading for synthesis of **5a** at 80°C.

Entry	Catalyst (mol %)	Yield (%)
**1**	10	traces
**2**	15	50
**3**	20	86
**4**	25	90
**5**	30	90
